# YAP regulates liver size and function

**DOI:** 10.1080/15384101.2017.1407390

**Published:** 2018-03-22

**Authors:** Norio Miyamura, Hiroshi Nishina

**Affiliations:** Department of Developmental and Regenerative Biology, Medical Research Institute, Tokyo Medical and Dental University (TMDU), 1–5–45 Yushima, Bunkyo-ku, Tokyo 113–8510, Japan

**Keywords:** Hepatocyte, liver sinusoidal endothelial cell, Kupffer cell, cell proliferation, cell migration, apoptosis, liver size, liver quality, Cdc42, Rac

The liver is one of the main detoxifying organs, removing waste and xenobiotics through metabolic conversion and biliary excretion. The waste and xenobiotics come from the gastrointestinal tract via the portal vein, and diffuse into small blood vessels known as hepatic sinusoids. Thus, the liver is constantly exposed to various stresses that can lead to tissue damage. The liver consists of several different cell types including hepatocytes, which have metabolizing and detoxifying abilities, liver sinusoidal endothelial cells (LSECs), which form the sinusoidal wall and cover the hepatocytes, and Kupffer cells, which are sinusoid-resident macrophages. Cellular stress in the liver leads to senescent, transformed, or damaged cells. These cells can impair tissue function or lead to tumorigenesis and therefore need to be eliminated and their loss compensated for by cell proliferation to maintain organ size. However, the molecular mechanisms that act to maintain three dimensional (3D) tissue and organ homeostasis during cellular stress are largely unknown.

The Hippo pathway regulates organ size and cancer formation by modulating cell proliferation and death via regulation of YAP activation [1–5]. Central to the Hippo pathway is a kinase cascade that activates the adaptor protein Mob and the protein kinase LATS. Activated LATS then phosphorylates the transcription co-activator YAP, and inhibits its activation by cytoplasmic retention. Unphosphorylated YAP translocates into the nucleus, interacts with the transcription factor TEAD, and induces target gene expression. Gene knockout of Hippo pathway components induces hepatomegaly and liver cancer in mice. Our colleague also reported that loss of Mob causes YAP activation and cancer formation in mouse liver[6,7]. Depletion of the YAP gene suppressed liver cancer formation in Mob knockout mice. Thus, the liver phenotypes caused by an impaired Hippo pathway are strongly dependent on YAP. Our group isolated a unique medaka fish mutant, *hirame (hir)*, which is sensitive to deformation by gravity. *hir* embryos display a markedly flattened body caused by mutation of YAP. We reported that YAP is essential for proper 3D body shape through regulation of cell tension[3]. In *Drosophila*, the cells with relatively lower fitness levels are eliminated from the tissue by a cell-cell interaction, which is called “cell competition”[4]. We found that active YAP-expressing mammalian epithelial (MDCK) cells are eliminated apically when the cells are surrounded by normal MDCK cells[5].

Our recent study has shown that YAP regulates the fate of hepatocytes by determining whether they proliferate to boost the organ's bulk or are degraded and removed[6]. This choice was shown to be dependent on whether the liver cells had been damaged, which deepens our understanding of how this organ maintains itself (Fig. 1). To examine how the Hippo pathway affects the fate of individual hepatocytes, we first established mosaic conditions by using hydrodynamic tail vein injection (HTVi) to introduce active YAP into mouse liver *in vivo*. We discovered that the fate of YAP-expressing hepatocytes changes from proliferation to migration/apoptosis depending on the status (healthy or damaged) of the liver. We also explored more directly whether adaptive immunity was involved in the loss of active YAP-expressing hepatocytes in immunodeficient NOG mice by HTVi. The numbers of hepatocytes steadily decreased also in NOG livers. Thus, the elimination of YAP-activated hepatocytes is regulated by a mechanism distinct from adaptive immunity-dependent senescence surveillance[7]. To identify the molecular mechanisms involved in YAP-mediated elimination of injured hepatocytes, we analysed gene expression profiles in mouse livers. Examination of the gene ontology annotations of genes upregulated in active YAP livers revealed the involvement of CDC42, which are small Rho family GTP proteins that regulate cytoskeleton organization and cell migration. We found that dominant negative CDC42 and Rac suppressed the elimination of YAP-activating hepatocytes. Thus, both CDC42 and Rac contribute to YAP-activated hepatocyte elimination. Furthermore, we identified the upstream regulators of CDC42 and Rac in hepatocytes expressing active YAP as Ect2 and Fgd3, which are guanine nucleotide exchange factors (GEF) for CDC42 and Rac. Finally, we found YAP activation plus HTVi or EtOH (i.e., tissue damage) induce Ect2 and Fgd3 upregulation, which triggers CDC42 and Rac activation in hepatocytes and drives their migration to sinusoids where they undergo Kupffer cell-mediated elimination. F-actin formation promotes YAP activation, and YAP regulates actin remodelling through the Rho GTPase activating protein[3]. Here we found that YAP induces the Rho GEFs Ect2 and Fgd3 in a manner dependent on an additional signal from LSECs. Thus, F-actin formation and YAP activation regulate each other through a feedback mechanism. In summary, YAP acts as a stress sensor that induces the elimination of injured cells to maintain tissue and organ homeostasis.

The Hippo pathway is constitutively activated and rapidly inactivates YAP by phosphorylation. Conversely, when the Hippo pathway is inactivated by stress, YAP immediately becomes unphosphorylated, translocates into the nucleus and induces target gene expression. Based on this, we deduce that YAP plays a role in an emergency stress response to maintain tissue homeostasis due to the elimination of injured cells. These findings demonstrate the complexity of cell fate determination mechanisms *in vivo*, and highlight a new role for YAP in tissue dynamics.

**Figure 1.**
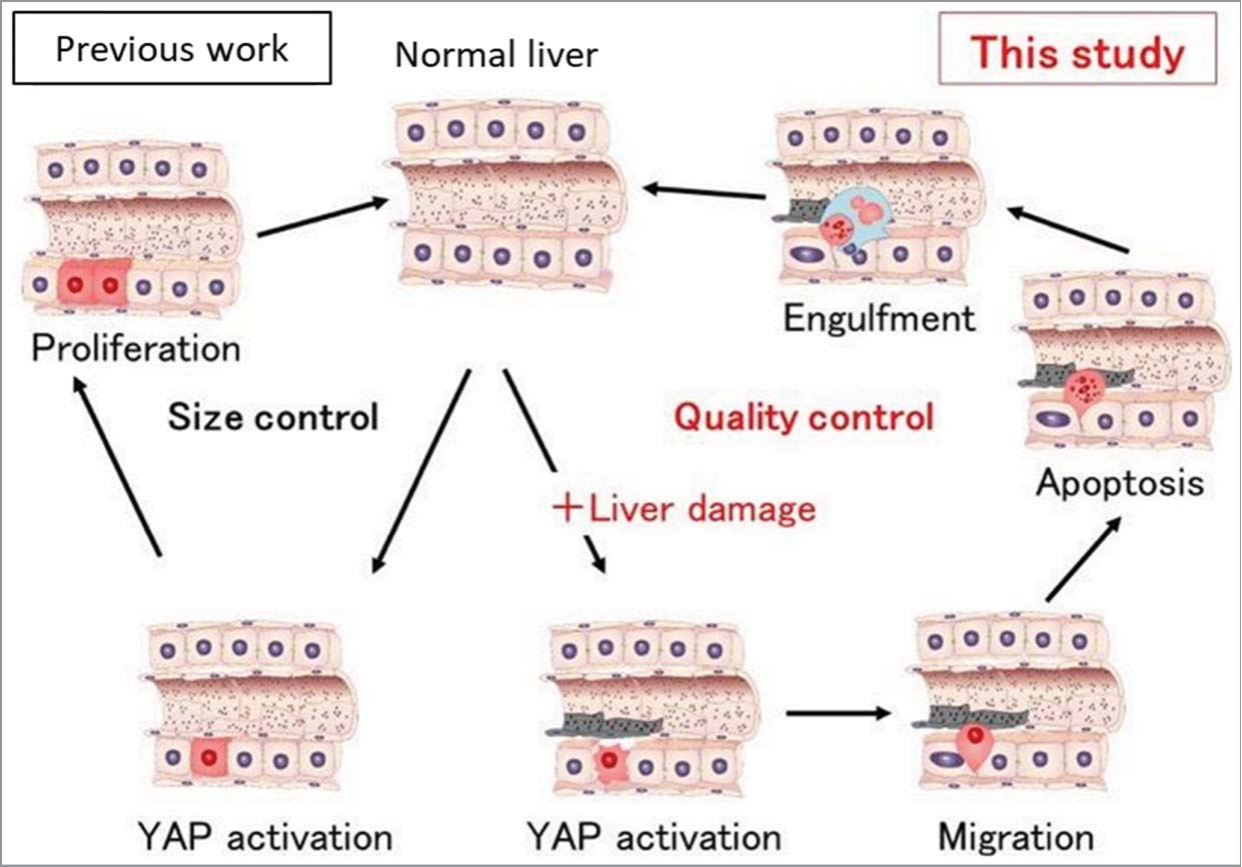
Active YAP (red cells) regulates liver size through hepatocyte proliferation (left; Previous work). In this study, we showed that active YAP selectively eliminates damaged hepatocytes (right). Hepatocytes expressing activated YAP in the presence of liver injury such as ethanol migrate into sinusoids, undergo apoptosis and are engulfed by Kupffer cells (blue).

## References

[1] Pan D. The hippo signaling pathway in development and cancer. Dev Cell. 2010;19:491–505. doi: 10.1016/j.devcel.2010.09.011. PMID: 20951342 20951342 PMC3124840

[2] Nishio M, Sugimachi K, Goto H, et al. Dysregulated YAP1/TAZ and TGF-β signaling mediate hepatocarcinogenesis in Mob1a/1b-deficient mice. Proc Natl Acad Sci. 2016;113:E71–E80. doi: 10.1073/pnas.1517188113. PMID: 26699479 26699479 PMC4711826

[3] Porazinski S, Wang H, Asaoka Y, et al. YAP is essential for tissue tension to ensure vertebrate 3D body shape. Nature. 2015;521:217–221. doi: 10.1038/nature14215. PMID: 25778702 25778702 PMC4720436

[4] Morata G, Martin FA Cell competition: the embrace of death. Dev Cell. 2007;13:1–2. doi: 10.1016/j.devcel.2007.06.002. PMID: 17609101 17609101

[5] Chiba T, Ishihara E, Miyamura N, et al. MDCK cells expressing constitutively active Yes-associated protein (YAP) undergo apical extrusion depending on neighboring cell status. Sci Rep. 2016; 6:28383. doi: 10.1038/srep28383. PMID: 27324860 27324860 PMC4914932

[6] Miyamura N, Hata S, Itoh T, et al. YAP determines the cell fate of injured mouse hepatocytes in vivo. Nat Commun. 2017;8:16017. doi: 10.1038/ncomms16017. PMID: 28681838 28681838 PMC5504293

[7] Kang TW, Yevsa T, Woller N, et al. Senescence surveillance of pre-malignant hepatocytes limits liver cancer development. Nature. 2011;479:547–551. doi: 10.1038/nature10599. PMID: 22080947 22080947

